# Assessment of Serum FABP‐4 Levels in Hypothyroidism Patients: A Comparative Analysis With a Control Group and Their Correlation With Liver Function Tests and Biochemical Factors

**DOI:** 10.1002/edm2.70011

**Published:** 2024-11-11

**Authors:** Mundher Mohammad Hamzeh Aldulaimi, Afsaneh Shafiei, Somayeh Ghorbani, Fatima Mohammadzadeh, Safoura Khajeniazi

**Affiliations:** ^1^ Metabolic Disorders Research Center Golestan University of Medical Sciences Gorgan Iran; ^2^ Department of Biochemistry and Biophysics Golestan University of Medical Sciences Gorgan Iran; ^3^ Cancer Research Center Golestan University of Medical Sciences Gorgan Iran

**Keywords:** adipokine, FABP‐4, hypothyroidism, lipid profile, thyroid hormones

## Abstract

**Background:**

The association of serum FABP4 and other biochemical‐related parameters is important to determine complications of hypothyroidism. This study aimed to evaluate serum FABP‐4 levels in hypothyroidism patients in comparison with a control group.

**Materials and Methods:**

Forty‐five patients with hypothyroidism and 45 healthy volunteers were included in this study. Liver function tests, thyroid hormones panel, lipid profile and serum FABP‐4 levels were measured and compared in both groups, and their correlations were analysed.

**Results:**

Serum FABP‐4 levels were significantly higher in patients with hypothyroidism compared to the control group (*p* = 0.002), and serum FABP‐4 level in males was higher than it in females (*p* = 0.022). There was a significant difference between patients with hypothyroidism and the control group in the levels of AST (*p* = 0.012). Moreover, serum FABP‐4 levels were negatively correlated with age (*p* = 0.016) and positively correlated with weight (*p* = 0.044).

**Conclusion:**

In our study, there was a notable increase in serum FABP‐4 concentration among hypothyroidism subjects. The data suggest that FABP‐4 could potentially be a superior diagnostic indicator for hypothyroidism when contrasted with a control cohort in future studies.

## Graphical abstract

The data suggest that FABP‐4 could potentially be a superior diagnostic indicator for hypothyroidism when contrasted with a control. Screening for Hypothyroidism, using FABP4 levels is a novel method which may be used to detect other hypothyroidism‐related diseases.

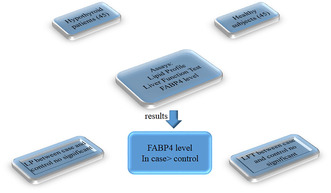

## 1 Introduction

Hypothyroidism has been recognised as a prevalent aetiology or a significant predisposing factor for numerous diseases, including obesity, cardiovascular diseases and cancer. Hypothyroidism tends to be linked with a slight increase in body weight, reduced thermogenesis and metabolic rate [1]. Clinical hypothyroidism is delineated by elevated serum levels of thyroid‐stimulating hormone and diminished serum levels of free peripheral thyroid hormones. Recent epidemiological studies have highlighted hypothyroidism as a paramount concern, presenting substantial threats to global public health. Many of these alterations in thyroid function are linked to changes in adipocytes. There is a notable interest in evaluating the correlation between adipocytokines, thyroid hormones and thyroid dysfunction [2, 3].

Considering the slight changes in the secretion of thyroid hormones leads to a broad range of homeostatic processes [4, 5]. A positive association between serum thyroid stimulating hormone (TSH) levels and body mass index (BMI) is proposed due to the link between thyroid dysfunction and weight changes [6, 7]. Thyroid hormones are crucial in regulating body metabolism including elevation in resting metabolic rate, boosting energy expenditure and inducing thermogenesis in adipose tissue [3]. Disruptions in thyroid function are associated with alterations in body weight, muscle mass and fat tissue. Metabolic syndrome is characterised by a combination of risk factors, such as visceral adiposity leading to insulin resistance and increasing susceptibility to type 2 diabetes, hypertension and dyslipidemia. Furthermore, an imbalance in hormonal levels of sex hormones, growth hormones and thyroid hormones may also contribute as a predisposing factor to the development of metabolic syndrome [8, 9].

An adipocyte is a metabolically active cell rather than just a reservoir for surplus energy. It generates numerous functional compounds with various physiological roles and substances [10]. One of these compounds known as adipocytokines which exerts endocrine, autocrine and paracrine effects on other tissues such as the liver and thyroid [11]. Adipocytes release various adipokines, including adiponectin, FABP4 and tumour necrosis factor (TNF)‐α [12, 13]. Little is understood regarding the potential impact of various adipokines on developing complications in thyroid dysfunction.

One specific adipokine is fatty acid binding protein 4 (FABP4), (also known as Adipocyte FABP [A‐FABP] or Adipocyte P2), a protein that transports fatty acids predominantly found in adipocytes and macrophages. This protein is crucial in fatty acids transportation from the bloodstream into tissues that utilise fatty acids as the energy source such as heart. Elevated levels of circulating FABP4 in humans are linked to insulin resistance and metabolic syndrome [14, 15]. The FABP4 gene has recently been identified as a common risk factor in a collaborative genome‐wide association study (GWAS) involving 500,000 individuals, focusing on CVD and type 2 diabetes [16, 17]. Despite the absence of signal peptides, FABP4 is discharged from adipocytes through an unconventional secretion mechanism in conjunction with lipolysis [18, 19, 20]. Increased levels of FABP4 in the bloodstream are correlated with some conditions such as obesity, dyslipidemia, atherosclerosis and renal dysfunction [21, 22]. In this study, we aimed to determine the serum levels of FABP4 in patients with hypothyroidism and compare them with those of the control group as well as their concentration correlation with the thyroid hormone panel, liver function tests and lipid profile.

## 2 Materials and Methods

In the context of a case–control study, the study group consisted of 45 patients between the ages of 19 and 64 years (14 males, 31 females) who were admitted to the Dezyani clinic with hypothyroidism and 45 people (11 males, 34 females) with no systemic disease who formed the healthy control (HC) group. Based on previous studies [23], the mean (standard deviation) of log (FABP4) in diabetes cases and healthy controls were 2.98 (0.42) and 2.73 (0.38), respectively. The sample size of 42 subjects in each group was calculated using G power software at a significance level of 0.05 and a power of 0.8. Finally, the required sample size considered 45 subjects in each group. The inclusion criteria for patients with hypothyroidism were as follows: (1) adult patients of hypothyroidism and healthy subjects were more than 18 years old, (2) all patients suffering hypothyroidism were new cases (without any treatment). The exclusion criteria for patients with hypothyroidism were as follows: (1) children and newborns, (2) pregnant and lactating women, (3) autoimmune patients or steroid ingestion (2 weeks prior to blood taking), (4) patients with inherited thyroid disease, (5) chronic disease like Diabetes mellitus and heart diseases, liver, cardiovascular, pancreas, biliary liver, diabetes mellitus and malignancies. All individuals slated for inclusion in the investigation were duly notified both orally and through written communication regarding the nature of the research. Furthermore, each participant provided an informed consent when they were enrolled.

### 2.1 Biochemical Analysis

Free triiodothyronine (fT3), free thyroxin (fT4) and thyroid‐stimulating hormone (TSH) were measured by direct enzymatic reaction ELISA (IDEALDIAG kit; Iran). Alanine aminotransferase (ALT), aspartate aminotransferase (AST), alkaline phosphatases (ALP) and gamma‐glutamyl transpeptidase (GGT) were measured by using the enzymatic methods (PARS AZMUN kit, Iran). Triglycerides (TG), low‐density lipoprotein‐cholesterol (LDL‐C), total cholesterol (TC) and high‐density lipoprotein‐cholesterol (HDL) by fully automated Mindray BS‐380. Serum FABP4 levels were measured by Sandwich ELISA at Metabolic Research Center (Human FABP4 ZelBio ELISA Kit; Germany).

### 2.2 Statistical Analysis

The Shapiro–Wilk test was used to determine whether continuous variables were normally distributed. Clinical variables are displayed for patients with hypothyroidism and healthy controls (HC) and are expressed as mean ± standard deviation. To compare the differences in clinical variables between the two groups, Student's *t*‐tests were for the comparison of variables with normal distribution, whereas the Mann–Whitney *U* test was used for variables that did not have a normal distribution. The correlation between two variables was carried out by Pearson or Spearman's correlation analysis. *p* < 0.05 was considered statistically significant.

## 3 Results

### 3.1 Demographic, Clinical and Liver Function Tests and Lipid Profile of Study Groups

This study was performed on 90 individuals (45 hypothyroid patients and 45 healthy subjects). It evaluated the association between FABP‐4 serum levels with demographics, liver function tests and lipid profile in hypothyroidism patients compared to healthy control. The study groups' demographics, liver function tests and lipid profiles are shown in Table 1. There was no statistically significant difference in age and sex between the groups (*p* = 0.952 and *p* = 0.483, respectively). There was no significant difference between the groups in the TG, Total‐Cho, VLDL, LDL and HDL (*p* = 0.534, *p* = 0.264, *p* = 0.913, *p* = 0.275, *p* = 0.370, respectively). In addition, there was no significant difference between patients with hypothyroidism and the healthy subjects in the levels GGT, ALT, ALP (*p* = 0.247, *p* = 0.294, *p* = 0.671), except AST (*p* = 0.012) (Table 1).

**TABLE 1:** Demographic and biochemical characteristics of study groups.

Variables	Hypothyroid (*n* = 45)	Healthy control (*n* = 45)	*p*
Age	46.00 ± 10.86	45.96 ± 11.00	0.952
Sex			
Female	31 (47.7%)	34 (52.3%)	0.483
Male	14 (56%)	11 (44%)	
TG (mg/dL)	136.07 ± 78.36	143.96 ± 80.22	0.534
Total‐Cho (mg/dL)	185.84 ± 38.74	177.09 ± 35.06	0.264
VLDL (mg/dL)	29.58 ± 21.26	28.23 ± 16.06	0.913
LDL (mg/dL)	98.2 ± 28.22	91.98 ± 25.37	0.275
HDL (mg/dL)	48.98 ± 13.55	46.02 ± 11.27	0.370
GGT (U/L)	27.06 ± 25.03	33.67 ± 29.70	0.247
ALP (U/L)	161.93 ± 47.87	154.51 ± 69.03	0.294
ALT (U/L)	21.84 ± 4.70	24.09 ± 9.90	0.671
AST (U/L)	17.82 ± 8.60	24.31 ± 14.009	0.012

### 3.2 Circulating FABP4 Levels in Patients and Control Group

**FIGURE 1:**
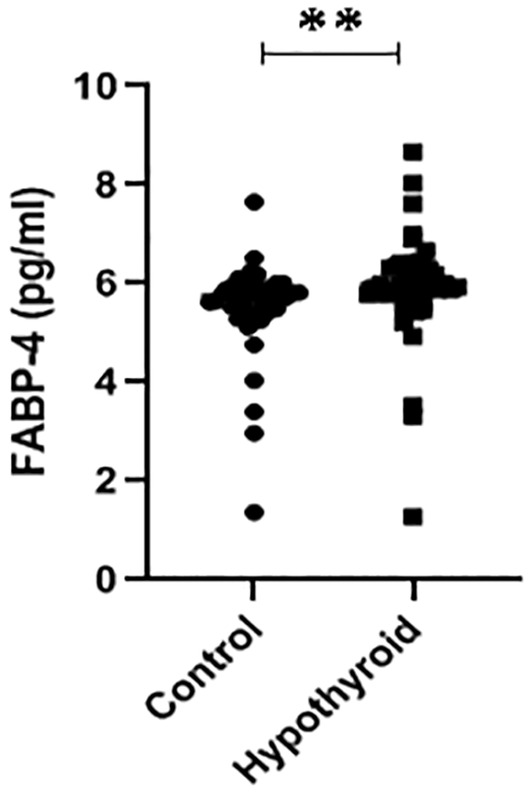
Levels of FABP4 in Control and Patients with hypothyroidism, **p < 0.05.

Serum FABP4 levels of patients with hypothyroidism and control group were 5.92 ± 1.13 (1.28–8.67) and 5.51 ± 0.96 (1.37–7.65) pg/mL, respectively. Serum FABP4 levels were higher in patients with Hypothyroidism than in HC (*p* = 0.002) (Figure 1). Moreover, serum FABP4 levels were higher in males with hypothyroidism than in females (*p* = 0.022). At the same time, there were no differences in serum FABP4 levels between males and females in the healthy control (Table 2).

**TABLE 2:** Comparison of serum FABP4 in females and males in studied groups.

Variables	Group	Female	Male	*p*	*p* ^a^
FABP4	Hypothyroid (*n* = 45)	5.737 ± 1.267	6.322 ± 0.602	0.022	0.002
	Healthy control (*n* = 45)	5.448 ± 0.956	5.702 ± 1.003	0.927	

^a^ Mann–Whitney test.

### 3.3 Correlation of Serum FABP4 With Thyroid Hormones Panel

There was a weak correlation but no significance between FABP4 and thyroid hormones panel in hypothyroidism and healthy control groups (Table 3).

**TABLE 3:** Correlation of FABP4 with thyroid hormones panel in case and control.

Thyroid panel	Hypothyroidism (*n* = 45)		Healthy control (*n* = 45)	
	*r*	*p*	*r*	*p*
T3	0.007	0.962	−0.198	0.192
T4	−0.131	0.391	−0.156	0.306
TSH	0.022	0.884	0.085	0.577

### 3.4 Correlation of Serum FABP4 With Liver Function

There was a weak correlation but no significance between FABP4 and liver function in hypothyroidism and healthy control groups (Table 4).

**TABLE 4:** Correlation of FABP4 with liver function in case and control.

Liver function	Hypothyroidism (*n* = 45)		Healthy control (*n* = 45)	
	*r*	*p*	*r*	*p*
GGT	−0.151	0.323	0.127	0.405
ALP	−0.151	0.323	0.019	0.902
ALT	0.019	0.899	0.122	0.423
AST	−0.001	0.993	0.139	0.363

### 3.5 Correlation of Serum FABP4 With Lipid Profile in Hypothyroidism and Healthy Control

In the healthy control, there was a significantly positive correlation between FABP4 and LDL (*r* = 0.361, *p* = 0.015), whereas there was a weak correlation but no significance FABP4 with TG, total‐Cho, VLDL and HDL. In the hypothyroidism group, there was a weak correlation but no significance FABP4 with TG, total‐Cho, VLDL, LDL and HDL (Table 5).

**TABLE 5:** Correlation of FABP4 with lipid profile in case and control.

Lipid profile	Hypothyroidism (*n* = 45)		Healthy control (*n* = 45)	
	*r*	*p*	*r*	*p*
TG	−0.123	0.421	0.177	0.246
Total‐Cho	−0.127	0.405	0.224	0.138
VLDL	−0.23	0.128	0.197	0.193
LDL	0.014	0.926	0.361	0.015
HDL	0.013	0.933	−0.081	0.597

### 3.6 Correlation Between FABP4 With Age and Weight in Hypothyroidism and Healthy Control

In hypothyroidism group, there was a significantly negative correlation FABP4 with age (*r* = −0.356, *p* = 0.016) and a significantly positive correlation FABP4 with weight (*r* = 0.301, *p* = 0.044) (Table 6).

**TABLE 6:** Correlation of FABP4 with age and weight in case and control.

Other parameters	Hypothyroidism (*n* = 45)		control (*n* = 45)	
	*r*	*p*	*r*	*p*
Age	−0.356*	0.016	0.035	0.820
Weight	0.301*	0.044	0.103	0.502

* Shows the correlation of FABP4 with age and weight in hypothyroidism is significant *p v*alue < 0.05.

## 4 Discussion

The excessive or absent expression of FABP4 has been demonstrated to play a role in various aspects of metabolic disorders and resulting negative consequences, including dyslipidemia, obesity, metabolic syndrome and atherosclerosis [24, 25, 26]. In the current study, we compared the difference in circulating FABP4 levels between 45 patients with hypothyroidism and 45 healthy controls. Then, we analysed the correlations between serum levels of FABP4 with thyroid hormone panel, liver function tests and lipid profile. Several studies have investigated the mechanisms that underlie the connections between FABP4 and thyroid hormone regulation. We displayed that patients with hypothyroidism were presented with higher serum FABP4 levels compared to the control group. In addition, we reported that increased serum levels of FABP4 in patients with hypothyroidism were not correlated with thyroid hormones panel. In contrast, in a previous study, an elevation in the concentration of FABP4 has been observed in individuals with subclinical and overt hypothyroidism [27]. Moreover, in our current study, serum levels of FABP4 were higher in males with hypothyroidism than it in females with hypothyroidism, and there was a significantly positive correlation between serum FABP4 and Weight. It is inconsistent with the results of Hong Wang et al. [28], who found that levels of serum FABP4 were increased in females with T2D compared to males. Additionally, a prior investigation conducted by Ibarretxe et al. demonstrated that serum FABP4 concentrations were influenced by gender, which is in line with our study [29]. The discrepancy in serum FABP4 levels between genders may be attributed to two main factors. Firstly, females typically possess higher body fat levels than males, given the strong association between serum FABP4 levels and adiposity. Secondly, androgens might play a role in explaining the gender disparity observed in serum FABP4 levels [28, 29, 30, 31].

In individuals classified as overweight or obese, a notable elevation in circulating FABP4 was identified in both male and female subjects. The robust positive correlation observed between serum FABP4 levels and various indicators of adiposity (such as BMI, waist circumference and fat percentage) strongly implies that adipose tissue is the main source of secreted FABP4 into the bloodstream [32, 33]. The divergence between sexes may be predominantly influenced by the higher fat percentage typically seen in women than men. Specifically, fat percentage emerged as a determining factor of the concentrations of circulating A‐FABP [34]. Moreover, variations in regional fat distribution between genders may play a role in the sexual dimorphism noted in A‐FABP concentrations among overweight or obese persons.

Prior investigations have revealed that Adipocyte fatty acid–binding protein (A‐FABP) plays a role in the development of cardiovascular and metabolic conditions associated with obesity [35]. The association between A‐FABP and the dysregulation of thyroid hormones remains unexplored and warrants further scrutiny. They revealed that increased serum levels of A‐FABP were correlated with diminished sensitivity to thyroid hormones in a euthyroid population, indicating a potential role of A‐FABP in facilitating communication between adipose tissue and the thyroid system [36].

Previous studies have indicated that the secretion of FABP4 as an adipokine has the direct capability to trigger the onset of insulin resistance and lipid metabolism [18, 19]. We documented that an increased level of FABP4 in patients with hypothyroidism was not correlated with lipid profile and liver function tests. Also, it has been documented that the administration of FABP4 externally induces endoplasmic reticulum stress in HepG2 liver cells which can potentially establish a connection between hepatic insulin resistance and metabolic dysfunction associated with obesity [37]. Their findings showed that the circulating FABP4 directly influenced liver malfunction, consequently leading to the progress of metabolic dysfunction‐associated fatty liver disease (MAFLD) [38]. Tanaka et al. reported the concentration of FABP4 is closely associated with FLI and serves as an autonomous predictor of MAFLD within a general population primarily including middle‐aged and elderly individuals [39].

Our study showed that there was a significant association between serum FABP4 concentrations and LDL‐c levels in the control group. Additionally, we noted that increased serum levels of FABP4 in patients with hypothyroidism were not correlated with LFT and lipid profile. Aimin Xu et al. [30, 40, 41] revealed that human subjects have a significant positive correlation between circulating A‐FABP levels and metabolic syndrome characteristics, including unfavourable lipid profiles (elevated serum triglycerides and LDL‐cholesterol and reduced HDL‐cholesterol). The physiological roles of circulating A‐FABP are still under investigation. A‐FABP can bind to various hydrophobic lipid molecules that are known to impact systemic metabolism and inflammation. It has been suggested that cytoplasmic A‐FABP plays a role in the internal movement and targeting of fatty acids within cells.

However, this localised function in adipocytes does not fully explain the metabolic influence of A‐FABP on distant targets like the liver and muscle [42, 43]. Circulating A‐FABP may be involved in transporting free fatty acids or other lipid hormones, which can subsequently regulate systemic insulin sensitivity and energy metabolism in the blood. Recent research has shown that retinol‐binding protein 4, another lipid‐binding protein produced in adipose tissue and liver, acts as an endocrine hormone contributing to insulin resistance and diabetes associated with obesity in mice [44, 45]. There are notable independent relationships between the level of FABP4 and both adiposity and high triglyceride levels. Women tend to possess subcutaneous fat more than men, whereas men exhibit higher abdominal (visceral) fat deposition levels, with distinct gene expression profiles in different fat depots. Further investigations are warranted to explore potential discrepancies in A‐FABP production across different fat depots in the human body [5].

## 5 Conclusion

Increased serum FABP4 levels were not closely associated with thyroid hormones panel, liver function tests or lipid profile in patients with hypothyroidism. This study explained the possible capacity that FABP4 could play as a potential biomarker for the prediction and prompt detection of patients with hypothyroidism.

## Author Contributions

Mundher Mohammad Hamzeh Aldulaimi contributed to the manuscript's data collection, review. Afsaneh Shafiei contributed to the study conception, the first draft, and the manuscript review. Somayeh Ghorbani performed the analysis of results. Fatima Mohammadzadeh cooperated in sample collection. Safoura Khajeniazi contributed to the conception and design of study, material preparation, supervision, writing the first draft of the manuscript, and review.

## Ethics Statement

This study was approved by the Ethics Committee of Golestan University of Medical Sciences, Gorgan, Iran (No. IR.GOUMS.REC.1402.118).

## Consent

Informed consent was obtained from all individual participants included in the study.

## Conflicts of Interest

The authors declare no conflicts of interest.

## Data Availability

The data set gathered and analysed during the current study is not publicly available but is available from the corresponding author upon reasonable request.
